# The Story of the Insane from Year to Year

**Published:** 1899-07-22

**Authors:** 


### THE STORY OF THE INSANE FROM YEAR TO YEAR.

(Eleventh Series.)

### THE NORWICH BOROUGH ASYLUM.

This asylum, unlike most others in the kingdom, was able to chronicle a decrease in numbers in 1897, there being 296 at the beginning of the year and 286 at the end of it. There were 80 first admissions, 16 readmissions, 30 recoveries, 22 discharged relieved, and 53 deaths. Calculated in the ordinary way, the recoveries stand at 35·71 per cent., and the deaths at 18·02 per cent. While the former is a little under the public asylum average, the latter is very much higher. Twenty-five of the deaths were from cerebral and spinal diseases, 3 from consumption, and 15 from senile decay. Intemperance in drink is the assigned cause of the insanity in 13 of the admissions, hereditary tendency in 17, and previous attacks in 20. The Norwich Borough is one of the smallest public asylums in England, and yet Dr. Harris tells us that he sent off 142 renewal certificates of insanity. It may be stated confidently that not one of these was necessary; but they have to be sent, and it may be imagined what a mass of extra work devolved on our medical superintendents on the passing of the Lunacy Act. The committee say they place unabated confidence in Dr. Harris. The average weekly cost per head is 9s. 2d.

### WARWICK COUNTY ASYLUM.

Here the number on the books on January 1st, 1897, was 870, and at the end of the year it had added exactly 100. There were 325 first admissions, 38 readmissions, 72 recoveries, 26 discharged relieved, and 110 deaths. Calculated in the usual way the recoveries stand at 30·3 per cent., and the deaths at 12 per cent.—the first being considerably below the county asylum average and the latter above it. Of the deaths 34 were caused by cerebral and spinal diseases, eight by pneumonia, and 27 by consumption. There were three accidental deaths, and one from enteric fever. The deaths from consumption seem very numerous, and Dr. Miller does not give any reason for this; but he makes the sensible suggestion that cases of consumption and severe diarrhœa should be treated in a suitable annexe, cut off from the main structure by a short lobby. Thirty-six of the year's admissions owed their insanity to moral causes, including fright, domestic troubles, &c.; and among physical causes intemperance in drink account for 23, and hereditary influence for 53. Asylums, like other and wiser communities, often have prevailing cries which are heard above all other requirements. At present the dominant note is the need of pathological research, and, like the refrain of a popular song, we hear it *ad nauseam*. Dr. Miller expresses his opinion, and with his opinion we fully concur, that the equipping and maintaining of a pathological laboratory so as to carry out systematic research under competent supervision, is rather too large an undertaking for one county, but the amalgamation of three or four neighbouring counties, and the maintenance of a laboratory under a committee appointed by them would be productive of practical benefit." Should Dr. Miller live to see this union of hearts in the Midlands we venture to predict for him a long life indeed; and long before the realisation of his and our wishes the London County Council, of which he speaks in this connection, will have spent thousands and thousands of pounds, and may be in a position to show an expectant world a high increase in the recovery rate traceable to these researches. We congratulate William Lewis (after 29 years' service), Martha Lewis (20 years), and George Hinson (20 years), on having received their pensions, and we wish them a long span of life and health in which to enjoy their repose. Whatever may be said about pensions in general there is no doubt whatever that asylum officials deserve them; and there is also no doubt that nothing will be productive of a better effect on the nursing staffs in asylums than a knowledge of the certainty of receiving pensions; and we therefore hope that the Lunacy Bill now before the House will become an Act before the end of the Session. We have consistently for the last ten years advocated this enactment, and we are pleased to see that we are within measurable distance of seeing the accomplishment of our wishes. The average weekly cost of maintenance per head is nearly 8s. 4d.

### THE BERKSHIRE COUNTY ASYLUM AT MOULSFORD.

There were in residence at the beginning of 1897 568 patients, and by the end of the year the numbers had risen to 623; but 26 of the year's admissions came from the Borough of Windsor. The total number of admissions was 162, and of these 136 were first admissions and 26 were readmissions. There were 40 recoveries, 10 discharged relieved, and 48 deaths. The percentage of recoveries calculated on the admissions stands at 24·6, but in reckoning this the transfers have been included. The death-rate was 8·13 per cent. of the average number resident. Of the deaths during the year 16 were caused by cerebral and spinal diseases, 6 by consumption, 4 by pneumonia, and 4 by bronchitis. Various moral causes account for 9 of the admissions, hereditary tendency alone or in combination with other causes accounts for 52, old age for 9, and general paralysis is put down for 9, but surely this is the form of insanity itself and not the cause. As the estimated accommodation is for 600 patients the numbers mentioned show that there must be some overcrowding, and it has been decided to increase the beds to 800. In the meantime the Boards of Guardians in the county have been requested to send to the asylum only those cases likely to be benefited by treatment. Dr. Murdoch points out that the recovery rate for the year is low compared with the average rate of English county asylums, and he truly states that "mere figures do not represent the best work of asylums." Dr. Murdoch may further console himself with the reflection that the higher the recovery rate in this generation the greater will be the amount of insanity in Berkshire in the next generation. We do not read in this report that any system of training the nurses for their important duties is in operation, and we hope this is merely an oversight of Dr. Murdoch's, or of ours, and that such training is given to staff. With two assistant medical officers and only about 600 patients there should be no difficulty in delivering lectures, practical instruction, &c., for a three years' course. The committee express "their continued satisfaction at the able manner in which all affairs of the asylum have been looked after and managed by Dr. Murdoch." The average weekly cost per head is 7s. 10½d. Out-county patients pay 14s., and there are eight private patients who pay 17s. 6d.

### THE LONDONDERRY DISTRICT ASYLUM.

Here the limits of accommodation are given at 344 beds, and yet we are informed that 452 patients were in residence on January 1st, 1897. As will be seen from these notices a similar state of things exists in many Irish asylums, and we have often wondered who is responsible for the delay in providing sufficient room for the wants of the district. During the year there were 77 first admissions, 20 readmissions, 43 recoveries, 13 discharged relieved, and 33 deaths. The recoveries stand at 44·3 per cent. of the admissions, and the deaths at 7·2 per cent. of the average number resident. Of the deaths 14 were caused by cerebral and spinal diseases, five by consumption, and five by inflammation of the lungs. As to the causes of insanity we find that 17 of the year's admissions were driven insane by such moral causes as domestic troubles, religion, and worry; 14 by intemperance in drink; and 24 by hereditary influence. Dr. Hetherington is to be congratulated on the efficiency of his nurses and attendants and on the duration of their service, for 26 have obtained the nursing certificate and 17 have had upwards of ten years' service. The annual cost per head was £22 9s. 9d.

### ROXBURGH DISTRICT ASYLUM.

Very few of the district asylums in Scotland would be looked on as "large," or even of moderate size, south of the Tweed. The one for Roxburgh, Berwick, and Selkirk contained only 277 patients at the beginning of the year, and 280 at the close of the year. There were 44 first admissions, 25 readmissions, 32 recoveries, and 23 deaths. These numbers show that 46·3 per cent. of the admissions recovered, and that 8·4 per cent. of the average number resident died. Of the deaths during the year only 10 were caused by cerebral diseases (of these only two were general paralytics) and two from consumption. Of the year's admissions moral causes account for the insanity in 18 instances, and physical causes are responsible in 106 instances. Of the former, adverse circumstances was the most potent factor, and of the latter, drink and heredity account respectively for 13 and 36. The asylum is being enlarged by the addition of a hospital for the female patients, and it would seem that the wants on the male side will soon necessitate a similar addition for male patients. It has already been decided to enlarge the general dining and recreation hall, and improvements in the laundry cannot be delayed much longer. We see, therefore, that even in small asylums in Scotland building operations never reach finality. Here also attendants and nurses are educated up to the standard of nursing required by the Medico-Psychological Society, and three passed the examination during the year. Dr. Johnstone notes that the work of the medical officers in this direction have borne fruit in the improved treatment of the patients; and he adds that this work has also "disseminated more enlightened views and skilled methods among the general community beyond the bounds of the asylum." The first statement is undoubted, and the second has much probability. The weekly cost of maintenance is almost 9s. 7d. per head.

